# Coherent intradimer dynamics in reaction centers of photosynthetic green bacterium *Chloroflexus aurantiacus*

**DOI:** 10.1038/s41598-019-57115-1

**Published:** 2020-01-14

**Authors:** Andrei G. Yakovlev, Vladimir A. Shuvalov

**Affiliations:** 1 Belozersky Institute of Physico-Chemical Biology, Lomonosov Moscow State University, Leninskie Gory, 119991 Moscow, Russia; 2 Institute of Basic Biological Problems, Russian Academy of Sciences, 142290 Pushchino, Moscow region Russia

**Keywords:** Biophysical chemistry, Bioenergetics, Supramolecular assembly

## Abstract

Early-time dynamics of absorbance changes (light minus dark) in the long-wavelength Q_y_ absorption band of bacteriochlorophyll dimer P of isolated reaction centers (RCs) from thermophilic green bacterium *Chloroflexus (Cfx.) aurantiacus* was studied by difference pump-probe spectroscopy with 18-fs resolution at cryogenic temperature. It was found that the stimulated emission spectrum gradually moves to the red on the ~100-fs time scale and subsequently oscillates with a major frequency of ~140 cm^−1^. By applying the non-secular Redfield theory and linear susceptibility theory, the coherent dynamics of the stimulated emission from the excited state of the primary electron donor, bacteriochlorophyll dimer P*, was modeled. The model showed the possibility of an extremely fast transition from the locally excited state P_1_* to the spectrally different excited state P_2_*. This transition is clearly seen in the kinetics of the stimulated emission at 880 and 945 nm, where mostly P_1_* and P_2_* states emit, respectively. These findings are similar to those obtained previously in RCs of the purple bacterium *Rhodobacter (Rba.) sphaeroides*. The assumption about the existence of the second excited state P_2_* helps to explain the complicated temporal behavior of the Δ*A* spectrum measured by pump-probe spectroscopy. It is interesting that, in spite of the strong coupling between the P_1_* and P_2_* states assumed in our model, the form of the coherent oscillations is mainly defined by pure vibrational coherence in the excited states. A possible nature of the P_2_* state is discussed.

## Introduction

Photosynthesis is a grandiose natural phenomenon of conversion of solar energy into stable energy of chemical compounds. Very shortly, absorption of light by pigment molecules creates an excited state, the energy of which is further used in a long sequence of biophysical and biochemical events. A first step in this sequence is charge separation occurring in special pigment-protein complexes, reaction centers (RCs). The conversion of light energy into free energy of charge-separated states occurs in RCs on a picosecond time scale.

The well-studied RCs of the photosynthetic purple bacterium *Rhodobacter (Rba.) sphaeroides* contain two exciton-coupled bacteriochlorophylls (P_A_ and P_B_), two bacteriochlorophylls (B_A_ and B_B_) in monomeric form, two bacteriopheophytins (H_A_ and H_B_), two quinones (Q_A_ and Q_B_) and a Fe atom. A spatial arrangement of the pigments is conserved by protein matrix. According to X-ray structure analysis, the pigments form two symmetrical branches, P_A_B_A_H_A_Q_A_ and P_B_B_B_H_B_Q_B_^1^. The P_A,B_ and Q_A,B_ are near the periplasmic and cytoplasmic sides of the photosynthetic membrane, respectively, while the B_A_,_B_ and H_A_,_B_ are in its central part. The Fe atom is between the Q_A_ and Q_B_. In *Rba. sphaeroides* RCs, a sequence of the primary electron-transfer reactions at room temperature can be written as P_A_ − 3 ps → B_A_ − 1 ps → H_A_ − 200 ps → Q_A_^2^. These reactions become two to three times faster at low temperatures. The quantum yield of the primary charge separation is slightly less than unity. In native RCs, the B-branch of the pigments is mostly blocked for charge separation. Thus, the primary charge separation leads to the formation of positive (P^+^) and negative (Q^−^) charges on opposite sides of the membrane.

The RCs from thermophilic green filamentous bacterium *Chloroflexus (Cfx.) aurantiacus* shows the similarity to the RCs of purple bacteria, (see for a review ref. ^3^). As in the purple bacteria, the dimeric bacteriochlorophyll is the primary electron donor, and the bacteriopheophytin molecule is the first electron acceptor in *Cfx. aurantiacus* RCs^4–6^. A three-dimensional structure of *Cfx. aurantiacus* RCs is unknown. However, spectroscopic and biochemical data together with exciton theory calculations suggest an existence of two pigment branches in *Cfx. aurantiacus* RCs like in RCs of purple bacteria^7,8^. The initial charge separation in the active A-branch of *Cfx. aurantiacus* RCs is slower than that in *Rba. sphaeroides* RCs^9,10^. For example, the decay of P* has a time constant of ~7 ps at 296 K. At 10 K, two main components for the P* decay have the time constants of 2 and 24 ps. The quantum yield of initial charge separation at 280 K^11^ and room temperature^12^ is near unity. The accessory bacteriochlorophyll molecule (B_A_) mediates electron transfer from P* to H_A_^12,13^. The time constant of H_A_^−^ → Q_A_ electron transfer in *Cfx. aurantiacus* RCs is ~320 ps at 280 K^14^. As in the purple bacteria RCs, the B-branch of the pigments is photochemically inactive in *Cfx. aurantiacus* RCs^11,12^. It should be noticed that protein and cofactor composition of *Cfx. aurantiacus* RCs (reviewed in ref. ^3^) differs from that of *Rba. sphaeroides* RCs. For example, Tyr M210 is replaced by Leu in *Cfx. aurantiacus* RCs, which probably reduces the rate of charge separation. Another interesting feature of *Cfx. aurantiacus* RCs is that a bacteriopheopytin molecule occupies the B_B_ binding site, and thus the inactive B-branch contains two bacteriopheopytins^5^.

As the primary electron donor, the dimer P plays a key role in charge separation. In *Rba. sphaeroides* RCs, the lower excitonic state of P has a strong Q_y_ absorption band near 865 nm at room temperature, whereas the upper excitonic state has a much weaker absorption band near 810 nm. Energy transfer from the upper to lower excitonic state is very fast (within 65 fs)^15^. At low temperature, the main absorption band of P is further red-shifted to ~895 nm. This red shift can be explained by coupling between the P* state and an optically dark charge-transfer state. This coupling was first discussed by Parson and Warshel^16,17^ and later elaborated by Renger^18^. In the Renger theory^18^, localization of the electronic states and, consequently, spectral positions of the absorption bands depends on temperature.

Another interesting property of the dimer P is that electron spin density distributes inhomogeneously between P_A_ and P_B_. Measurements of the hyperfine coupling constants in the oxidized P^+^ state revealed a shift of the electron π-spin density towards P_A_^19^. The ratio of P_A_: P_B_ electron density varies from 2: 1 in wild type RCs to 5: 1 in mutant RCs in which the residue Leu M160 was replaced with His^20^. Also, the molecular orbital calculations for the P* state showed that the electron density is strongly shifted from P_A_ to P_B_ (~1:3)^21^. The strong shift of the electron spin density in the P can be considered as a partial charge separation, i.e. a formation of the intermediate charge-transfer state P_A_^δ+^P_B_^δ−^. Several optical properties of RCs can be better explained by taking into account the intradimer charge transfer. For example, it was shown that the Stark effect in the Q_y_ band of *Rba. sphaeroides* RCs may indicate internal charge separation in the P* state^22^. Also, spectra of linear and circular dichroism of the P may be interpreted in favor of the partial intradimer charge separation^16^. A modeling of excitons in the excited state of *Blastochloris viridis* RCs led to a conclusion about a charge separation in the first excited state^23^. The same conclusion was done by modeling of the optical spectra of magnesium-bacteriochlorin dimers^24^. *Ab initio* quantum chemical calculations for molecular orbitals of the P in *Rba. sphaeroides* RCs are more consistent with the existence of the P_A_^δ+^P_B_^δ−^ state^25^. It was concluded that intradimer charge separation may be one of the reasons for unidirectional electron transfer along the active A-branch^25^. The state with P^+^ character mixed with the P* state was found in the YM210W mutant RCs by pump-dump-probe spectroscopy^26^. The ps lifetime of this charge-transfer intermediate was found to be of the order of the P* lifetime. Transient mid-infrared spectroscopy in the region of 1000–1600 cm^−1^ revealed a signal in the Δ*A* (light – dark) spectrum of *Rba. sphaeroides* RCs raised with a delay of 200 fs after an excitation of the P^27^. This observation was discussed in terms of internal conversion of the initial P* state to the different charge-transfer excited state which is accompanied by a change in the P* electronic structure (or in the P* electron-vibrational coupling). Dynamics of the mixed P*(P_A_^δ+^P_B_^δ−^) and P*(P_A_^δ+^B_A_^δ−^) states were studied in native and heterodimer *Rba. sphaeroides* RCs by time-resolved spectroscopy in the near-infrared region of 1060–1130 nm^28^. It was found that the absorption of the P_A_^δ+^ is delayed by 120–180 fs with respect to the P bleaching. This delay is close to the 200-fs delay observed in ref. ^27^.

Nuclear motions are involved in the primary charge separation in RCs. These motions can be visualized when a broadband coherent excitation of the RCs creates a vibrational wavepacket. The wavepacket oscillatory motion inside the P* potential well results in damped oscillations in the Δ*A* kinetics^29–31^. A Fourier transform spectrum of the oscillations shows several low-frequency vibrational modes, similar to those found by the resonance Raman spectroscopy^32,33^ and hole-burning experiments^34^. Molecular dynamics (MD) calculations show that some low-frequency vibrational modes strongly couple with the primary charge separation in *Rba. sphaeroides* RCs^35,36^. For example, the P and axial histidine HisM202 collectively move (HisM202 also rotates) with the mean frequency of 100 cm^−1^. This complicated motion are coupled to the P* dynamics and contributed to the intradimer charge separation. The collective motion of the interstitial water and its nearest environment with the 30–35 cm^−1^ frequency also influences on charge separation in RCs. The theoretical results of Eisenmayer *et al*.^35,36^ are in line with experimental^37^ and theoretical^38^ findings obtained earlier.

It was shown both theoretically^39^ and experimentally^15^ that electronic coherence can be another source of pronounced oscillations in kinetics. Sugawara *et al*.^39^ have shown that the coupling of two electronic states should not be small for producing distinct electronic oscillations. As a result of the simultaneous coherent excitation of the upper and lower excitonic states of the P, Arnett *et al*.^15^ observed a strongly damped (within 35 fs) oscillatory behavior of anisotropy with a frequency that exactly corresponded to the energy gap (~600 cm^−1^) between the two states. Notice that oscillations in the kinetics of different states of RCs may have an incoherent nature. By using the stochastic Langevin equation, the possibility of incoherent oscillations in the P_A_^+^B_A_^−^ state was demonstrated for the case of the protein relaxation-controlled reaction^40^. A simple kinetic model was used to illustrate the creation of intense incoherent oscillations in the stimulated emission from P* and the absorption of B_A_^−^ ^41^. A combination of MD methods and the stochastic Langevin approach was used to study the influence of the protein dynamics on the formation of the P_L_^+^P_M_^−^ dipole^42^. It was demonstrated by Milanovsky *et al*.^42^ that elastic vibrations should be taken into account to explain the phenomenon of excitation energy trapping in the P.

Herein, early-time dynamics of the P* state of *Cfx. aurantiacus* RCs was studied by difference pump-probe spectroscopy with 18-fs resolution at 80 K. The dynamical Stokes shift and coherent oscillations were observed in the P* stimulated emission of these RCs (as well as in *Rba. sphaeroides* RCs^29–31^) on a fs time-scale. The Redfield theory was used to explain the phenomena observed in the P* stimulated emission. The goal of the work was to find more arguments in favor of the existence of two different electronic states within the P* state. We modeled the oscillatory behavior of the time-resolved spectral profiles of the P* stimulated emission by the density matrix formalism and the transient linear susceptibility theory. We have demonstrated that the kinetics near 880 nm (the blue-most part of the P* stimulated emission) indicate most clearly that the initially excited state, P_1_*, relaxes very fast to the different excited state, P_2_*. This relaxation involves both population and vibrational coherence transfer. At longer wavelengths, intense oscillations in the stimulated emission of the P* state masked a spectral exhibition of the P_1_* → P_2_*electronic relaxation.

## Results and Discussion

**Figure 1:**
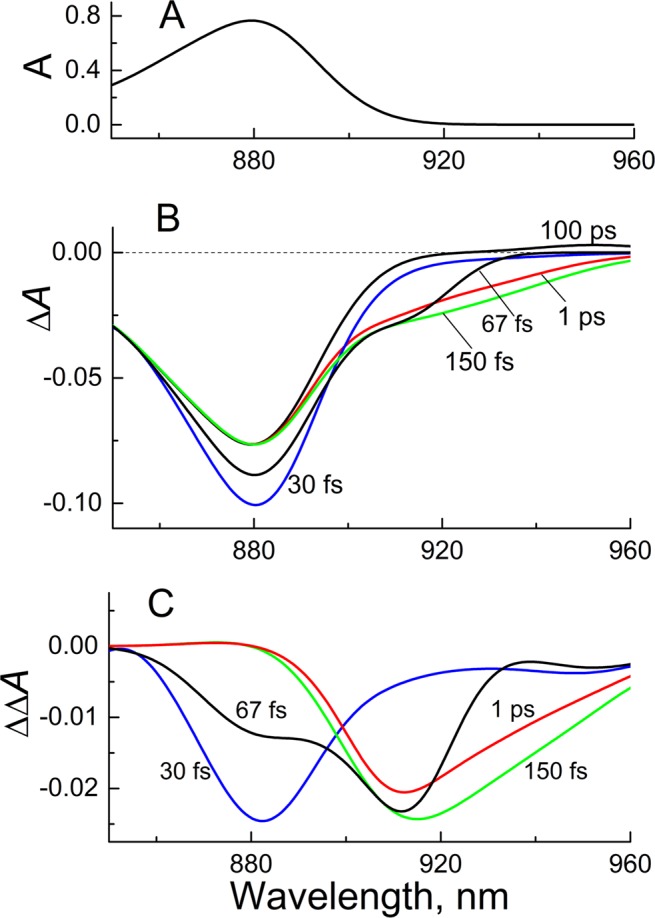
Absorption spectra of *Cfx. aurantiacus* RCs at 80 K. (**A**) Ground-state absorption spectrum in the Q_y_ P band absorption region. (**B**) Difference (light – dark) absorption spectra at delay times of 30, 67, 150, 1000 fs and 100 ps. Zero delay time corresponds to the center of the pump pulse. (**C**) Double difference absorption spectra calculated by subtraction of the 100-ps spectrum from the early-time spectra in panel (B).

**Figure 2:**
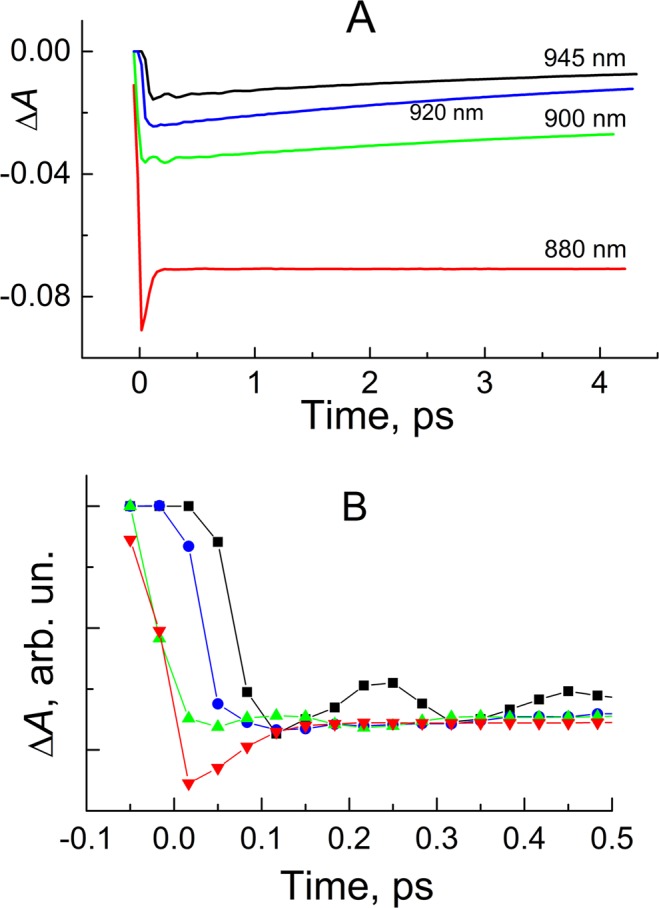
(**A**) Kinetics of *Cfx. aurantiacus* RCs at 880, 900, 920 and 945 nm at 80 K. (**B**) As (**A**) but normalized kinetics at 0–0.5 ps delay times.

Figure 1 shows ground-state and time-resolved absorption spectra of *Cfx. aurantiacus* RCs at 80 K. The near IR absorption band centered at ~880 nm is the Q_y_ P band. Photobleaching of the Q_y_ absorption band of P and red-shifted stimulated emission from P* are two main processes contributing to the Δ*A* spectrum of *Cfx. aurantiacus* RCs in the near IR region. At 30-fs delay time the photobleaching and stimulated emission centered near 880 nm. At 67 fs a new band appeared at ~910 nm, and the 880-nm band relaxed in part. The band at 910 nm is generally attributed to the pure P* → P stimulated emission. At 150 fs the stimulated emission band broadened to the red, and the 880-nm band further relaxed. On a ps time scale the overall amplitude of the stimulated emission band gradually decreased due to decay of the P* state into charge-separated states. As a result, at 100 ps the stimulated emission completely disappeared in the Δ*A* signal. Thus, the 100-ps Δ*A* signal is a pure absorption of P. To extract a major part of the P* stimulated emission spectrum, we subtracted the 100-ps signal from the fs Δ*A* signals (Fig. 1C). The P* → P stimulated emission gradually shifted from ~882 to ~915 nm within ~150 fs. This shift is clearly seen in Δ*A* kinetics at selected wavelengths (Fig. 2). For example, an initial negative rise in the 945-nm kinetics appeared later than that in the 880-nm kinetics by ~100 fs. Similar dynamical red shift was observed in the stimulated emission of *Rba. sphaeroides* RCs^28,29^.

**Figure 3:**
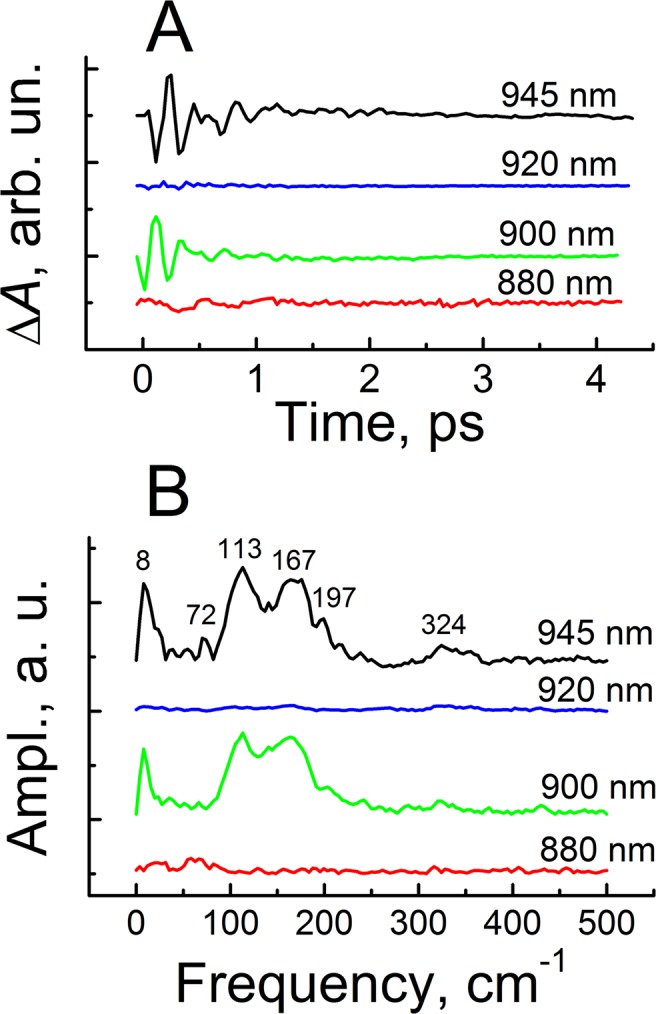
(**A**) Oscillatory parts of the kinetics in Fig. 2 obtained by fitting the data to exponential functions. (**B**) Fourier transform of the oscillatory parts in panel (A).

Also, the Δ*A* kinetics contained small oscillations with opposite phases on the red (945 nm) and blue (900 nm) sides of the stimulated emission band. The oscillations vanished in the center of the band (~920 nm). The stimulated emission from P* is a major source of coherent phenomena in the Δ*A* signal. For further analysis, we fitted the kinetics to exponential functions and subtracted the fit from the kinetics. A good fit of the kinetics measured at λ > 900 nm was achieved by a sum of 3.5 ± 0.2-ps (65 ± 2%), 15 ± 1.5-ps (30 ± 2%) and 70 ± 10-fs (5 ± 1%) exponents and an instrumental function. The best fit provided a near-symmetry form of the residual (kinetics minus fit) with respect to Y = 0 line (Fig. 3A). The scatter in the exponential parameters of the fit corresponded to ±5%-deviation of the residual from that of the best fit. The time constants and the percentage of the exponential components could be varied within the shown boundaries without noticeable aggravation of the fit. The fit was slightly varied with wavelength within the boundaries in a complicated manner. The 880-nm kinetics was fitted by 70-fs exponent, a constant and an instrumental function. Figure 3A shows pure oscillations of the Δ*A* kinetics. Major parts of the 900 and 945-nm oscillations (with a period of ~200 fs) are damped within ~1 ps. However, very small non-regular pikes are seen in the 945-nm kinetics at ~2 ps. Also, very small noise-like oscillations are observed in the 880-nm kinetics. The Fourier transform analysis of the oscillatory parts of the Δ*A* kinetics measured at 900 and 945 nm revealed several overlapped bands at 8, 72, 113, 167, 197 and 324 cm^−1^ (Fig. 3B). A center of gravity of these modes is at ~140 cm^−1^ (period ~220 fs). The oscillatory picture can be qualitatively fitted by single damped cosine with this frequency. The experimental data presented here are in good accordance with previously reported ps^9^ and fs^43^ results on *Cfx. aurantiacus* RCs. A nature of the oscillations in *Cfx. aurantiacus* RCs is still under debate. Very similar oscillations were found in *Rba. sphaeroides* RCs^29,37^. In the *Rba. sphaeroides* RCs, similarity of the oscillation frequencies to those found by the resonance Raman spectroscopy^32,33^ and hole-burning experiments^34^ led to the conclusion of their vibrational origin. Unfortunately, the resonance Raman and hole-burning data on *Cfx. aurantiacus* RCs are not available now.

**Figure 4:**
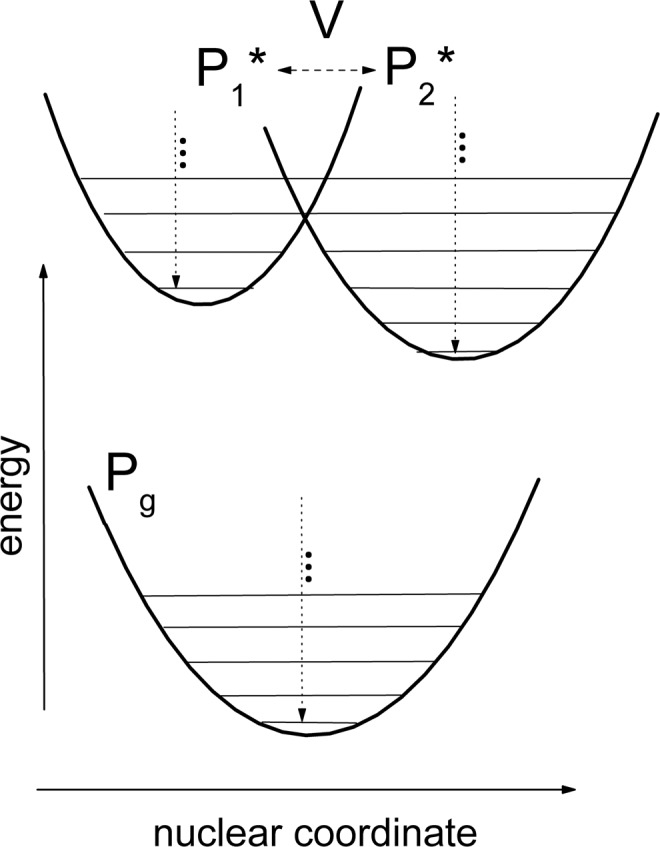
A scheme of energy levels of the ground (P_g_) and excited (P_1_* and P_2_*) states. Vibrational relaxation is shown by dotted arrows. The coupling between P_1_* and P_2_* is shown by a dashed double arrow. The energy shift between P_1_* and P_2_* terms is taken as 2$\hslash$*ω*_*vib*_. The dimensionless coordinate shift of the P_1_* and P_2_* terms with respect to the P_g_ term is taken as *Δ*_1_ = −1 (the Huang-Rhys factor *S*_1_ = 0.5) and *Δ*_2_ = 1.4 (*S*_2_ = 1), respectively.

Theoretical simulation of the experimental data presented here was based on the generalized linear response theory and the Redfield density matrix formalism in one-mode approach (see M&M section for brief and Supplement for full theoretical background). We considered two electronically coupled excited states, P_1_* and P_2_* (Fig. 4), instead of the single P* state by the following reasons. First, the observed spectral changes are too fast (~100 fs) to be explained by vibrational relaxation in the single P* state (usually ~several hundreds of fs). Energy redistribution between different vibrational modes due to nonlinear resonances or pure dephasing of modes that are strongly coupled to P* → P transition may has such high rate, but a clear experimental indication on these phenomena in *Cfx. aurantiacus* RCs are not available now. Second, the dynamical red shift of the stimulated emission seems too large (~30 nm) to be explained by any vibrational process inside the single P* state. It is accepted that in RCs most of the high-frequency (several hundreds of cm^−1^) vibrational modes that are the main contributors to the P absorption/emission spectra have the small Huang-Rhys factor *S*^17^. Only few low-frequency modes have *S* *≥* 1. With the small *S*, the vibrational relaxation mainly causes the change in spectral form, but not in positions of the P* and P spectra on the frequency scale. Therefore, the simplest way to explain our experimental observations is to assume an existence of two excited states, P_1_* and P_2_*. It is clear that the simple one-mode approximation is far from providing a detailed quantitative modeling of the experiment. On the other hand, mathematics of multi- mode non-markovian approach (for example, hierarxical equations of motion) is extremely difficult.

**Figure 5:**
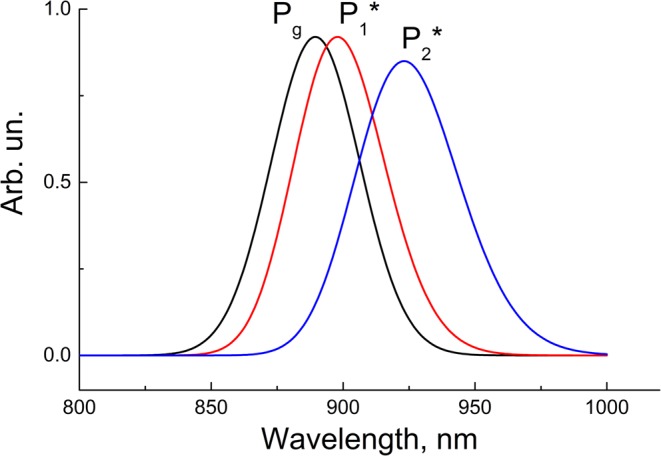
Calculated absorption spectrum (P_g_) and steady-state emission spectra (P_1_* and P_2_*) at low temperature. The bandwidth of Gaussian vibrational bands is taken as 470 cm^−1^ (FWHM). The 0 → 0 transition frequency is taken as 11,190 cm^−1^ for P_1_* → P_g_ transition and 10,940 cm^−1^ for P_2_* → P_g_ transition. The vibrational frequency is taken as 125 cm^−1^.

**Figure 6:**
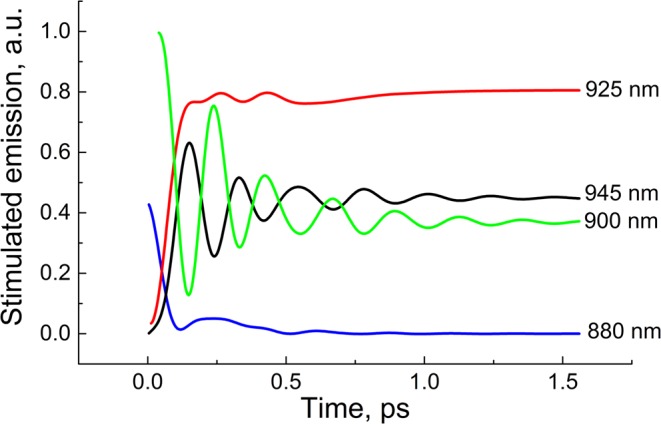
Calculated kinetics of the stimulated emission at 880 (mostly the P_1_* state), 900, 925 and 945 nm (mostly the P_2_* state). Pure electronic coupling between P_1_* and P_2_* is taken as *J* = 250 cm^−1^. The rate constant of the 1 → 0 vibrational transition is 6 ps^−1^. For details of the other parameters, see the text and the legend in Figs. 4 and 5.

The calculation of the steady-state emission spectra for the P_1_* and P_2_* states shows a strong overlap between them (Fig. 5). To reveal the dynamics of the pure P_1_* or P_2_* states, we calculated emission kinetics at the short-wavelength tail of the P_1_* emission (880 nm) and the long-wavelength tail of the P_2_* emission (945 nm) (Fig. 6). The kinetics at 880 nm, reflecting mostly the P_1_* dynamics, show a very fast (~100 fs) decrease of the initial population and subsequent small and slow near-zero variations. This kinetics look like exponential relaxation, but actually it is close to overdamped oscillations. In contrast, the kinetics at 945 nm (mostly the P_2_* dynamics) show a very fast population of the P_2_* state and subsequent strong oscillations with a frequency ~*ω*_*vib*_. These oscillations are damped on a ps time scale. In the middle spectral area from 890 to 930 nm, the dynamics of the stimulated emission reflect a complicated mixture of the P_1_* and P_2_* dynamics (Fig. 6). For example, the kinetics at 900 nm have pronounced oscillations with the same frequency ~*ω*_*vib*_, but their phase is opposite to the phase of the oscillations at 945 nm. In contrast, the kinetics at 925 nm show a fast initial rise and almost complete absence of oscillation. Theoretical results shown in Fig. 6 are in accordance with experimental ones (Figs. 2 and 3) as well as with other experiments^28–30,43^. It is clear from Fig. 6 that a strong coupling between the two excited states leads to the extremely rapid transfer of the population and vibrational coherence from the initially excited P_1_* state to the P_2_*. Backward transfer is impeded by fast thermal equilibration in the vibrational manifolds. At a smaller rate constant of vibrational equilibration, the backward coherent transfer becomes more pronounced, which results in oscillations in the P_1_* state at 880 nm (Supplementary Fig. S2).

In order to separate contributions of the vibrational and vibronic coherences to the whole dynamics of the excited states, we calculated the kinetics at 880 and 945 nm for the case of zero vibrational (pure vibronic) coherence (Supplementary Fig. S3). In this case, the initial depopulation of the P_1_* state and population of the P_2_* state proceed noticeably more slowly, and the oscillations in both kinetics are very small. This means that, in spite of a large value for the pure electronic coupling *J* (250 cm^−1^) used in the calculations, the contribution of the vibronic coherence to the oscillation feature is smaller than that of the vibrational coherence.

As mentioned above, both the population and coherence of the P_2_* state are zero at *t* = 0 in our calculations. In Supplementary Figure S4, the kinetics at 880 and 945 nm are shown for the case when an initial vibrational wavepacket is presented in the P_2_* state too. Except for a moderate increase of the oscillation amplitude, the kinetics are quite similar to those shown in Fig. 5. This proves that the backward (P_2_* → P_1_*) transfer of vibrational coherence is small in our model.

The Huang-Rhys factor *S* is of great importance in our model, because an overlap of the vibrational wavefunctions strongly depends on *S*^44^. If the P_1_* and P_2_* terms are shifted in the opposite direction with respect to the ground term, then the factor *S*_12_ responsible for the overlap of the P_1_* and P_2_* vibrational wavefunctions can be defined as *S*_12_ = (√*S*_1_ + √*S*_2_)^2^. For the dimensionless coordinate shifts *Δ*_1_ = −1   (*S*_1_ = 0.5) and *Δ*_2_ = 1.4 (*S*_2_ = 1) used in our model, *S*_12_ = 2.9. At this value of *S*_12_, the maximal overlap is achieved between the lowest vibrational level with *n* = 0 from the P_1_* term and the levels with *n* = 1, 2, and 3 from the P_2_* term. In contrast, shifting P_1_* and P_2_* in the same direction with respect to the ground term results in the much smaller value of the *S*_12_ = (√*S*_2_ − √*S*_1_)^2^ = 0.09. For a small *S*_12_ ≪ 1, the maximal overlap is achieved for vibrational levels with *n* = 0 from both excited terms. This seems to be optimal only if the P_1_* and P_2_* states have the same energy, which is not the case. Furthermore, a small *S*_12_ leads to a small overall rate of the P_1_* → P_2_* reaction. These arguments are proved by a calculation of the kinetics for the case of *S*_12_ = 0.09 (Supplementary Fig. S5). After a moderate initial decrease, the kinetics at 880 nm (mostly the P_1_* state) show an oscillatory behavior around a nonzero value that is not consistent with experiments. The *S* value used in our model is larger than usually assumed for photosynthetic pigments. As we mentioned above, it is accepted in the literature that in *Rba. sphaeroides* RCs only few low-frequency (≤100 cm^−1^) modes have *S* *≥* 1^17^. These modes considerably contribute to absorption and emission spectra of P and play an important role in the formation of long-lived coherent oscillations. No information on *S* is available for vibrational modes of *Cfx. aurantiacus* RCs. Probably the low-frequency modes of *Cfx. aurantiacus* RCs also have the large *S* factor. As we argued above, the main reason for the choice of the large *S* in our model was a necessity to explain a high rate of the transition between the two states which are shifted in energy with respect to each other.

The electronic coupling *J* is another important parameter in our model. In order to obtain a high rate of the P_1_* → P_2_* reaction, we used *J* = 250 cm^−1^ in the calculations. For *S*_12_ = 2.9, the maximal value of the overlap integrals is of the order of 0.2, which provides a value of the coupling energy of ~50 cm^−1^. For a smaller *J* (40 cm^−1^), the population transfer from the initially excited P_1_* state to the P_2_* state proceeds on a picosecond time scale and is accompanied by oscillations in both the states (Supplementary Fig. S6). For a very large *J* = 800 cm^−1^, no further acceleration of the P_1_* → P_2_* reaction is observed in the kinetics at 880 and 945 nm (Supplementary Fig. S7). In the last case, the high frequency vibronic oscillations are clearly seen in the P_1_* kinetics. Notice that the both cases shown in Supplementary Figures S6 and S7 are not consistent with experiments.

The value of the energy gap between the P_1_* and P_2_* states is of great importance too. In our model, we assumed that the P_2_* term is downshifted by 2$\hslash$*ω*_*vib*_ with respect to the P_1_* term. This assumption provides a reasonable accordance of the calculated spectra and kinetics with experimental ones. For a smaller shift ~$\hslash$*ω*_*vib*_, a slow subpicosecond component is observed in the calculated kinetics at 880 and 945 nm (Supplementary Fig. S8), that is not proved by experiments. For zero energy gap, equilibrated populations of the P_1_* and P_2_* states are of the same order.

Also, we studied a “secular” variant of our model, in which zero values of *R*_*nm,n’m’*_ and zero couplings between non-isoenergetic levels were assumed. In this case, the calculated kinetics show a poor consistency with experiments because of the small amplitude of the oscillations and slow component of the kinetics (Supplementary Fig. S9). These calculations show the importance of non-secular processes for better explanation of the experimental results.

The principal concept of our model is the assumption about the existence of two different states in P*. To examine the validity of this assumption, we simulated the coherent phenomena in the model with the single excited state of P. We found that the oscillatory kinetics of both models are similar in the spectral area of λ ≥ 900 nm and are quite different at shorter wavelength of λ < 900 nm. In both models, the oscillations in the kinetics at 900 and 945 nm have opposite phases and similar amplitudes, while the oscillations in the vicinity of 925 nm are much smaller in amplitude (by more than 20 times). At 880 nm, the model with the single P* state resulted in the kinetics with clear oscillations that look like decreased and slightly distorted oscillations at 900 nm. This result is in contradiction with the low temperature experimental data (ref. 29 and this work) and with the P_1_* → P_2_* model, both of which show non-oscillatory kinetics at 870–880 nm. At room temperature, the oscillations are presented in the experimental kinetics at 870 nm too, probably because of the increased width of the P* emission spectrum^28^. Thus, we have shown that the sequential P_1_* → P_2_* model is more realistic than the P* model at least in the case of low temperature.

In spite of a great amount of various theoretical studies on primary photosynthetic processes, there is a lack of works which cover very early processes inside the dimer P. A theoretical study of the intradimer events has been done in terms of classical Markus theory with time-dependent rate constants^41^. In this simple illustrative approach, the existence of two excited states of P with close energies was postulated. For explanation of the oscillations with opposite phases at 905 and 940 nm, an external modulation of the forward and backward rate constants was assumed. It was found that the amplitude of the modulation should be greater than the amplitude of thermal fluctuations in order to fit the experimentally observed oscillations. A source of the modulation was revealed by quantum MD simulations^35,36^. These *ab initio* calculations showed that some low-frequency vibrational modes are strongly coupled with primary charge separation in *Rba. sphaeroides* RCs. One of them, the mode with a frequency of ~100 cm^−1^ represents the collective motion of P and a rotation of the axial histidine HisM202. The participation of this mode in intradimer charge transfer and its coupling to the dynamics of the excited state of P were demonstrated by Eisenmayer *et al*.^35,36^. Another mode with a frequency of ~30 cm^−1^ is associated with the collective motion of the interstitial water and its hydrogen bond network. The influence of the 130 and 32 cm^−1^ vibrational modes was studied earlier by Novoderezhkin *et al*.^38^ with the theory based on density matrix formalism. The model of Novoderezhkin *et al*.^38^ included two mixed diabatic states (one excited state, P*, and one charge-separated state, P^+^B_A_^−^) that were strongly coupled with two collective vibrational modes. It was demonstrated in ref. ^38^ that the nuclear wavepacket is initially created in the P* state and then is transferred by small portions to the P^+^B_A_^−^ state. The coherence transfer is driven by the two modes with frequencies of 130 and 32 cm^−1^.

The density matrix method together with MD simulations were applied to study the primary charge separation between P* and B_A_^17^. The model included five effective low-frequency vibrational modes with frequencies of 30, 70, 125, 175 and 235 cm^−1^. The frequencies, displacements and microscopic time constants were obtained from MD simulations by the dispersed-polaron approach, whereas the electron-transfer dynamics were controlled by a stochastic Liouville equation. Oscillations of the energy gap between the P* and P^+^B_A_^−^ states resulted in out-of-phase oscillations of the populations of these states. An inverse temperature dependence of the reaction rate was found to be based on the assumption that an electron is transferred faster than the vibrational relaxation is completed. In the model of Parson and Warshel^17^, electronic coupling between the P* and P^+^B_A_^−^ states was assumed to be ~10–20 cm^−1^, i.e. less than the vibrational energies. It seems that the analogous coupling between the different states of P* may be greater.

In a recent theoretical study, the primary photosynthetic reactions were simulated by MD methods together with the phenomenological Langevin approach^42^. The authors calculated a dielectric response upon the initial charge separation inside the dimer P_L_P_M_ leading to the formation of the P_L_^+^P_M_^−^ dipole. They found that the neighboring environment of the dimer (such as axial ligands His172, His202 and α-helices) plays an important role in the dynamics of the formation of the P_L_^+^P_M_^−^ dipole. The protein dynamics around P_L_ and P_M_ were found to be asymmetric with higher amplitude of vibrations around P_L_. The main frequencies of the vibrations were found in the range of 30–120 cm^−1^, i.e. the frequency range of the oscillations revealed in the stimulated emission from P* by the pump-probe spectroscopy. It follows from ref. ^42^ that the low-frequency coherent oscillations may originate from both extradimer and intradimer motions.

The nature of the P_2_* state as well as the origin of the P_1_* → P_2_* relaxation are not yet completely understood. Internal conversion and vibrational energy redistribution are the processes providing fast intramolecular energy transfer and dissipation of excess energy. These processes may be responsible for the population of the P_2_* state. The internal conversion can be described in terms of a conical intersection between the potential energy surfaces of the states^45,46^. By examining the fast relaxation of the upper excitonic state P*_+_ into the lower state P*_−_, Chang *et al*.^45^ concluded that the 560 cm^−1^ vibrational mode may participate as a promoting mode in the reduction of the energy gap between these two states. Anharmonic couplings between vibrational modes are a possible reason for the vibrational energy redistribution. Both diabatic and adiabatic mechanisms of the internal conversion may be responsible for the fast electronic relaxation. The fast (~100 fs) incoherent transfer between the conically intersected diabatic potential surfaces of the excited states of chlorophyll *a* was theoretically studied by Dong *et al*.^47^. It seems that the adiabatic mechanism of the P_1_* → P_2_* transition may not be able to provide a very high transition rate^48^.

A strong coherence could be another possible mechanism of the fast P_1_* → P_2_* transition. By using quantum theory with density matrix formalism, Sugawara *et al*.^39^ have studied the effect of both vibrational and vibronic coherences on primary charge separation. They have shown that in the case of weak electronic coupling between the excited and charge-separated states the form of the oscillations in the stimulated emission is mostly determined by the vibrational coherence. But, if the electronic coupling is strong enough, a nonlinear mixing of the vibronic and vibrational frequencies results in a noticeable distortion of the oscillations. In the case of strong electronic coupling and a high rate of vibrational equilibration, the population of the initially excited state can be coherently transferred to another excited state in a nearly irreversible manner. The rate of this transfer can be estimated as ~2 *V*/$\hslash$, where *V* is the electronic coupling. In terms of wavepacket motion, the irreversible coherent transfer can be rationalized as a one-step transition of the vibrational wavepacket during its first (and only) approach to the splitting area of the potential surfaces of the P_1_* and P_2_* states. If the energy split between the surfaces (~2 *V*) is greater than the wavepacket energy, than the probability of wavepacket transition is ~1. In the opposite case of weak coupling between the two reactant states (for example, P* and P^+^B_A_^−^), the wavepacket leaks through the splitting area from one state to another in small portions^38^.

One can speculate that P_2_* is the state with charge-transfer character, i.e. the state P_A_^δ+^P_B_^δ−^ with partial charge separation between the two bacteriochlorophylls constituting the dimer P. The same assumption about the initially excited P_1_* state cannot be excluded from consideration, although the experimental data^27^ are more consistent with a small charge-transfer contribution in the P_1_* state. The question of whether the sub-100 fs time constant of the P_1_* → P_2_* relaxation reflects the dynamics of intradimer charge separation or not is still open. It seems more grounded to discuss the very early intradimer events in terms of the mixed P* (P_A_^δ+^P_B_^δ−^) state rather than separated states. The dynamics of this mixed state should include both the processes of intradimer energy and charge transfer which may occur on the same time scale. According to quantum-chemical calculations^24^, additional coupling to charge-transfer states (P_A_^δ+^P_B_^δ−^ or P_A_^δ−^P_B_^δ+^) led to a lowering of the energy of the excited state of P. In our model, the electronic energy of the P_2_* state was taken to be less than that of P_1_* by 250 cm^−1^ in order to properly explain the transient red shift of the stimulated emission from the excited state of P. One may see, to some extent, a possible analogy between this red shift and the known dual fluorescence from two excited states of electron donor-acceptor molecules such as 4-aminobenzontriles and *N*-phenylpyrroles^49^. In these molecules, an intramolecular charge-transfer state with a larger dipole moment is produced from a locally excited state by conformational changes on a picosecond time scale. On the other hand, the sub-100 fs electronic relaxation of the excited state of P obtained by our modeling seems to be too fast for any conformational motion. Recent MD simulations show that intradimer charge separation leads to significant changes of the polarization around the dimer P^42^. The reaction field dynamics of this polarization include a very fast response with a characteristic time of <50 fs and a magnitude of 50–70 mV followed by a slower oscillatory behavior. So, the time scale of the fast polarization effects in the calculations of Milanovsky *et al*.^42^ is similar to that of P_1_* → P_2_* relaxation in our model.

Very recently, two dimentional electronic spectroscopy technique with 17-fs pulses was applied to mutant RCs from the purple bacterium *Rba. sphaeroides* at 77 K^50^. This work strongly supported a well-known sequential scheme of charge separation, in which the very first step is a formation of the P_A_^δ+^P_B_^δ−^ state. It was shown that the electronic coherence with a lifetime 100–200 fs and the vibrational coherence with a lifetime ~2 ps are accompanied the coherent P* → P_A_^δ+^P_B_^δ−^ transition. The dynamical red shift and oscillations in the stimulated emission are the most important features of the coherent intradimer charge separation. The electronic coherence is responsible for the red shift and the first intense peak of the oscillations. It was concluded that the coherence play an important role in optimization of the primary charge separation in the purple bacterium RCs. Our observations and interpretations on RCs from the green bacterium *Cfx. aurantiacus* are in line with the work of van Grondelle and co-workers^50^. Based on their results, we can say more definitely that the P_2_* state in our model can be the P*(P_A_^δ+^P_B_^δ−^) state with charge-transfer character.

## Conclusions

Fs dynamics of the P* → P stimulated emission in isolated RCs of photosynthetic green bacterium *Cfx. aurantiacus* was studied by difference (light minus dark) pump-probe spectroscopy at cryogenic temperature. It was shown that the stimulated emission spectrum exhibits a pronounced Stokes shift from 880 to 915 nm within ~150 fs followed by oscillations with a mean frequency of ~140 cm^−1^. Previously similar phenomena were extensively studied in RCs of the purple bacterium *Rba. sphaeroides*^28–31,37^. We modeled these phenomena by applying the transient linear susceptibility theory and the Redfield density matrix formalism to the P_1_* → P_2_* scheme of energy transfer between locally excited (P_1_*) and relaxed (P_2_*) states. Our simulations of the coherent dynamics of the P* → P stimulated emission are consistent with experimental data under the following realistic assumptions: (i) energy downshift of the P_2_* state with respect to the P_1_* state, (ii) strong coupling between the P_1_* and P_2_* states, (iii) fast vibrational relaxation in the both excited states. With these assumptions, a sub-100 fs coherent transition occurs from the initially excited P_1_* state to the P_2_* state. This transfer is almost irreversible. The modeling showed that the sequential scheme with two spectrally different excited states of P is preferable for the explanation of experimental data. A form of the coherent oscillations is mainly defined by pure vibrational coherence in the excited states in spite of a pronounced electronic coupling between them assumed in our model. A detailed atomistic study of intramolecular energy and charge transfer processes in dimer P is necessary for deeper understanding of the very early photosynthetic events.

## Materials and Methods

### Isolation and purification of reaction centers

RCs of *Cfx. aurantiacus* were prepared according to ref. ^51,52^ with minor modifications. Shortly, cells were sonicated in 50 mM Tris-HCl buffer (pH 8.5) and, after removal of unbroken cells and debris membranes, were isolated by centrifugation at 100,000 × *g*. Membranes (A865 = 15 cm^−1^) were incubated with 1% LDAO in 50 mM Tris-HCl buffer (pH 8.5) in the presence of 50 mM NaCl at 37 °C for 1 hour, followed by centrifugation at 100,000 × *g* for 2.5 hours (sodium dithionite was added to the incubation mixture to a final concentration of 10 mM in order to exclude the possibility of a partial destructive oxidation of RCs). RCs were purified from the resulting supernatant by repeated anion exchange chromatography on DEAE cellulose DE52 columns and eluted with 50 mM Tris-HCl, pH 8.5/0.1% LDAO/60 mM NaCl buffer.

For time-resolved measurements, the detergent LDAO was exchanged for Triton X-100 in the buffer solutions by repeated cycles of diluting samples with 50 mM Tris-HCl, pH 8.5/0.05% Triton X-100 and reconcentrating on a membrane in a pressure cell under argon gas. The samples were desalted during this procedure. Low-temperature (80 K) measurements were performed on the samples containing 65% (v/v) glycerol. The absorbance of the RCs at 860 nm was adjusted to 0.5 in a 1-mm optical path-length cell at room temperature. Sodium dithionite (5 mM) was added to keep RCs in the state PB_A_H_A_Q_A_^−^.

### Time-resolved femtosecond spectroscopy

Standard pump-probe spectroscopy technique was used for time-resolved measurements^53^. Details of our experimental setup are available in ref. ^37,43^. Fs transient absorption-difference measurements were carried out with a Tsunami Ti:sapphire femtosecond laser (Spectra Physics, USA). Fs pulses were amplified by an eight-pass Ti:sapphire amplifier (Avesta, Russian Federation) followed by a continuum generator, a pump-probe scheme, and an optical multichannel analyzer (Oriel, France). The operating frequency was 40 Hz. The durations of pump and probe pulses were 18 fs. Spectrally broad pump pulses were centered at 870 nm. The delay time between pump and probe pulses could be set with an accuracy of 1 fs. The temporal dispersion of the probe pulses in the spectral region of 860–960 nm was compensated by home-made four-prism compensator to less than 30 fs. The residual chirp was measured by the spectral band bleach of the green glass filter (ZC-10, LOMO, St. Petersburg). The time-resolved spectra and kinetic time scales were corrected accordingly. Transient absorption-difference spectra were obtained by averaging 7,000–10,000 measurements at each delay time. The accuracy of absorbance measurements was 3 × 10^−5^ units of optical density. The kinetics of absorbance changes (Δ*A*) were plotted at selected wavelengths. The nonoscillating fits were found mathematically and subtracted from the kinetics. The oscillatory residuals were analyzed by Fourier transformation.

### Model

The theoretical background is available in the Supplement. Here we briefly list a model description. We considered three vibrational manifolds: one manifold for an electronic ground state P_g_ and two manifolds for excited states P_1_* and P_2_* (Fig. 6). We made the following standard assumptions: (i) the duration of the pump and probe pulses is short in comparison with the system time scale, (ii) the pump and probe pulses are not overlapped in time, (iii) the intensity of the pulses is weak enough. We considered a case of very low temperatures: *k*_*B*_*T* ≪ $\hslash$*ω*_*vib*_. According to the generalized linear response theory^39^, in the Condon approximation, the stimulated emission of the system can be written as:

1 $$E(\omega ,t) \sim {\sum }_{{\rm{nm}}}{\rm{Re}}[{F}_{nm}(\omega ){\rho }_{nm}(t)],$$

where *ρ*_*nm*_(*t*) is the density matrix element of the system and *F*_*nm*_(*ω*) is the spectral function. According to the density matrix formalism based on the Redfield theory, the time evolution of the system can be written as^46^:

2 $$d{\rho }_{nm}/dt=-\,(i{\omega }_{nm}+{R}_{nm,nm}){\rho }_{nm}-\sum {\sum }_{n^{\prime} m^{\prime} }{R}_{nm,n^{\prime} m^{\prime} }{\rho }_{n^{\prime} m^{\prime} }-i/\hslash \,{\sum }_{k,l}({V}_{nk}{\rho }_{km}-{\rho }_{nl}{V}_{lm})$$

here $\hslash$*ω*_*nm*_ is the energy difference between levels numbered by *n* and *m*. The matrix elements of the Redfield relaxation tensor *R* represent the processes of population decay and transition, dephasing, coherence transfer and coupling between populations and coherences. The matrix elements of *V* represent the coupling between the P_1_* and P_2_* manifolds. A*t t* = 0, initial values of the matrix elements *ρ*_*nm*_(0) depend on the absorption spectrum of P and the energy of the pump pulse. The spectral function *F*_*nm*_*(ω*) can be written as:

3 $${F}_{nm}(\omega )={\sum }_{{\rm{p}}}\langle em|gp\rangle \langle gp|en\rangle \,L({\omega }_{el}-\omega +(m-p){\omega }_{vib})$$

here ‹*em*|*gp*› and ‹*gp*|*en*› are the vibrational overlap integrals (*g* denotes ground state, and *e* denotes the P_1_* or P_2_* excited states), and *L* is a line shape function.

## Supplementary information

Dataset 1.

## Data Availability

The datasets related to the current study are available from the corresponding author on reasonable request.
