# Supplementary material for: Coherent intradimer dynamics in reaction centers of photosynthetic green bacterium Chloroflexus aurantiacus
Source: Sci Rep. 2020 Jan 14;10:228. doi: 10.1038/s41598-019-57115-1 (PMC6959224; doi:10.1038/s41598-019-57115-1)
Supplement: Supplementary file 1 — Dataset 1. [file 41598_2019_57115_MOESM1_ESM.pdf]

# Supplement

# Coherent intradimer dynamics in reaction centers of photosynthetic green bacterium *Chloroflexus aurantiacus*

**Andrei G. Yakovlev[1,*], Vladimir A. Shuvalov[1,2]**

[1] *Belozersky Institute of Physico-Chemical Biology, Lomonosov Moscow State University, Leninskie Gory, 119991, Moscow, Russia. Fax: +07-495-939-31-81, tel.: +07-495-939-53-63, e-mail: yakov@genebee.msu.ru*

[2] *Institute of Basic Biological Problems, Russian Academy of Sciences, 142290, Pushchino, Moscow region, Russia. Fax: +07-496-779-05-32, e-mail: shuvalva@gmail.com*

* Author for correspondence

## 1. Theory

Based on experimental data reported in the MS, we focused on the excited state of P and used a one-mode approach in our model. We considered three vibrational manifolds: one manifold for an electronic ground state $P_g$ and two manifolds for excited states $P_1$* and $P_2$* (Fig. S1). The excited states are electronically coupled with each other. According to the model of displaced harmonic oscillators, we assumed a parabolic potential surface for each electronic state. In Figure 1, the $P_1$* and $P_2$* potential surfaces are shifted in the opposite directions along a nuclear coordinate with respect to the $P_g$ surface. We found this configuration to be optimal (see the MS). For simplicity, we assumed that an energy downshift for the $P_2$* state with respect to the $P_1$* state is equal to the integer numbers of vibrational quanta $\hbar\omega_{vib}$. This assumption seems to be of little importance if the bandwidth of the vibrational levels is of the same order as $\hbar\omega_{vib}$. We assumed that direct optical excitation of the $P_2$* state is impossible. When the system is probed, both the $P_1$* and $P_2$* states produce stimulated emission. Next, we made the following standard assumptions: i) the duration of the pump and probe pulses is short in comparison with the system time scale, ii) the pump and probe pulses are not overlapped in time, iii) the intensity of the pulses is weak enough. Our model does not include charge-transfer states $P^+B_A^-$ or $P^+H_A^-$, so the model can be directly applied to describe the early P* dynamics on a time scale that is much shorter than a time constant of charge separation. This time scale is of several ps for the slow *Rba. sphaeroides* mutants, such as YM210W and YM210L[1], and of less than 1 ps for the *Cfx. aurantiacus*[2] and native *Rba. sphaeroides*[3] RCs. We considered a case of very low temperatures: $k_BT \ll \hbar\omega_{vib}$.

According to the generalized linear response theory[4-8], in the Condon approximation, the stimulated emission of the system can be written as:

$$E(\omega,t) \sim \sum_{nm} \mathrm{Re}[F_{nm}(\omega)\,\rho_{nm}(t)], \qquad (1)$$

where $\rho_{nm}(t)$ is the density matrix element of the system and $F_{nm}(\omega)$ is the spectral function. If indexes $n$ and $m$ belong to one and the same manifold ($P_1$* or $P_2$*), then the diagonal ($n = m$) and off-diagonal ($n \neq m$) matrix elements $\rho_{nm}(t)$ represent populations and vibrational coherences, respectively. If indexes $n$ and $m$ belong to the different manifolds, then the matrix elements $\rho_{nm}(t)$ represent vibronic coherences. According to the density matrix formalism based on the Redfield theory, the time evolution of the system can be written as[9]:

$$d\rho_{nm}/dt = -(i\omega_{nm} + R_{nm,nm})\,\rho_{nm} - \sum_{n'}\sum_{m'} R_{nm,n'm'}\,\rho_{n'm'} - i/\hbar \sum_{k,l}(V_{nk}\,\rho_{km} - \rho_{nl}\,V_{lm}) \qquad (2)$$

Here $\hbar\omega_{nm}$ is the energy difference between levels numbered by $n$ and $m$ ($\omega_{nm} = 0$ if $n = m$). The matrix elements of the Redfield relaxation tensor $R$ represent the processes of population decay and transition, dephasing, coherence transfer and coupling between populations and coherences[10]. Analytical expressions for those processes can be found within the step-ladder model[8]. Notice that all of the $R$ elements depend on a single parameter, the rate constant of the $1 \rightarrow 0$ vibrational transition. The matrix elements of $V$ represent the coupling between the $P_1$* and $P_2$* manifolds. Each of them is a product of the pure electronic coupling $J$ and the overlap integral. In the case of displaced harmonic oscillators, an analytical form of the overlaps is well known[11]. For populations and vibrational coherences, $k = l$ and both $k$ and $l$ indexes belong to another manifold (to $P_2$* if $n$ and $m$ belong to $P_1$* and *vice versa*). For vibronic coherences, $k = m'$ and $l = n'$. At $t = 0$, initial values of the matrix elements $\rho_{nm}(0)$ depend on the absorption spectrum of P and the energy of the pump pulse. We assumed that the initial populations and vibrational coherences are created in the state $P_1$* at $t = 0$, while the state $P_2$* is initially empty.

The spectral function $F_{nm}(\omega)$ can be written as[6]:

$$F_{nm}(\omega) = \sum_{p} \langle em|gp\rangle\langle gp|en\rangle L(\omega_{el} - \omega + (m - p)\omega_{vib}) \qquad (3)$$

Here $\langle em|gp\rangle$ and $\langle gp|en\rangle$ are the vibrational overlap integrals ($g$ denotes ground state, and $e$ denotes the $P_1$* or $P_2$* excited states), and $L$ is a complex Lorentzian line shape function. For more realism, we assumed the $L$ function to be Gaussian.

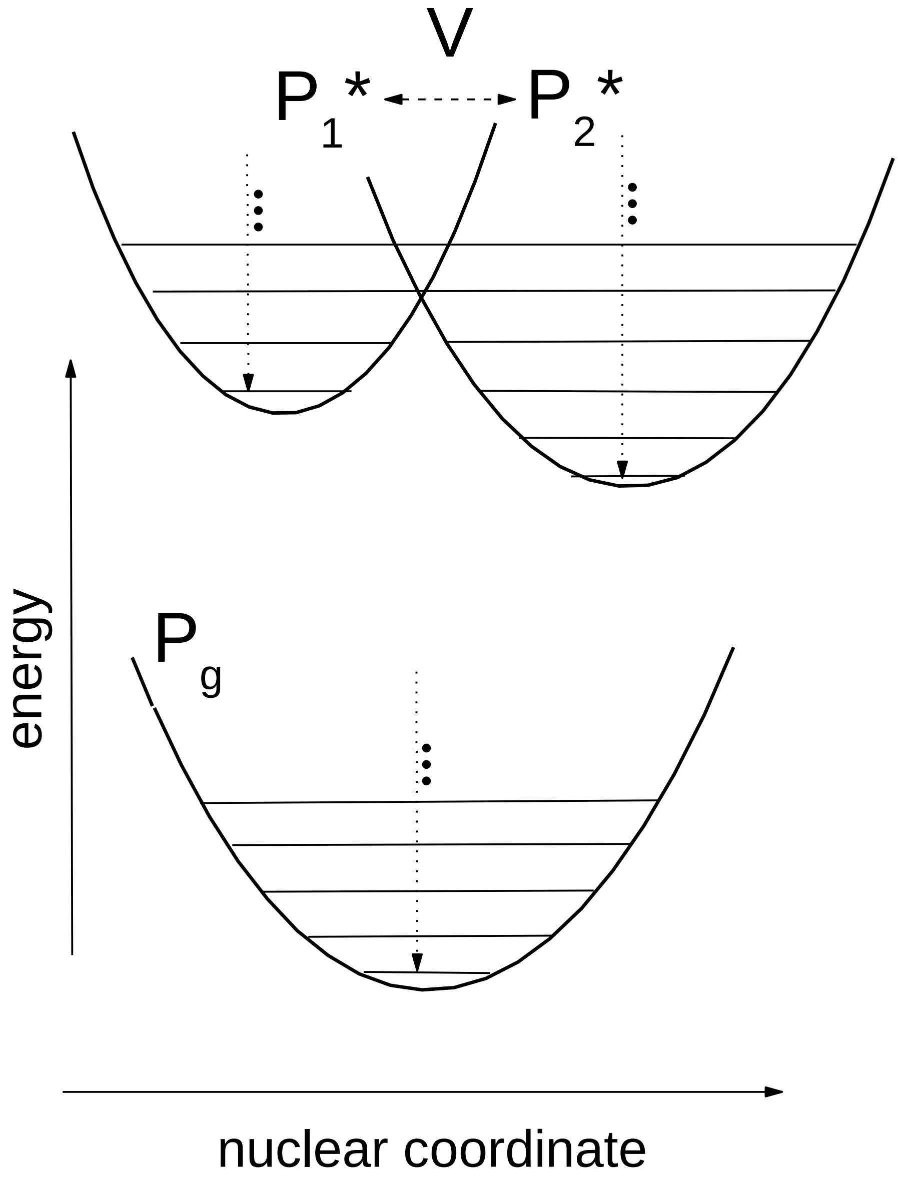


**Figure S1.** A scheme of energy levels of the ground ($P_g$) and excited ($P_1$* and $P_2$*) states. Vibrational relaxation is shown by dotted arrows. The coupling between $P_1$* and $P_2$* is shown by a dashed double arrow. The energy shift between $P_1$* and $P_2$* terms is taken as $2\hbar\omega_{vib}$. The dimensionless coordinate shift of the $P_1$* and $P_2$* terms with respect to the $P_g$ term is taken as $\Delta_1 = -1$ (the Huang-Rhys factor $S_1 = 0.5$) and $\Delta_2 = 1.4$ ($S_2 = 1$), respectively.

## 2. Calculations

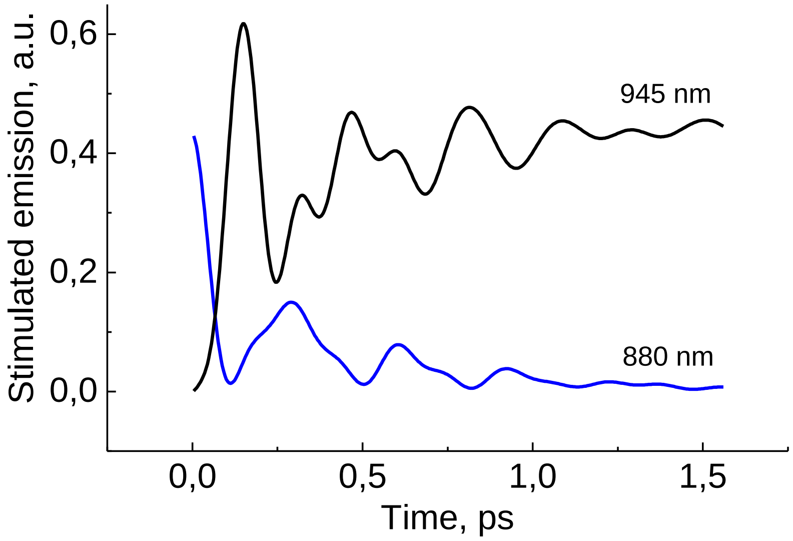


**Figure S2.** Calculated kinetics of the stimulated emission at 870 (mostly the $P_1$* state) and 960 nm (mostly the $P_2$* state). Pure electronic coupling between $P_1$* and $P_2$* is taken as $J = 250$ cm$^{-1}$. The rate constant of the 1→0 vibrational transition is 2 ps$^{-1}$. For details of the other parameters, see the main text and the legend in Figure S1 and 4.

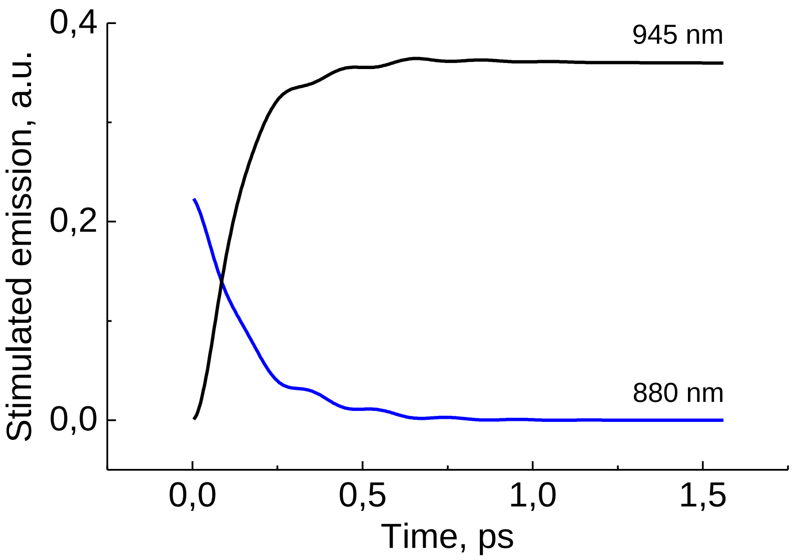


**Figure S3.** As Figure S2 but with zero vibrational coherence (pure vibronic coherence). For details of the other parameters, see the main text and the legends in Figures S1, S2 and 4.

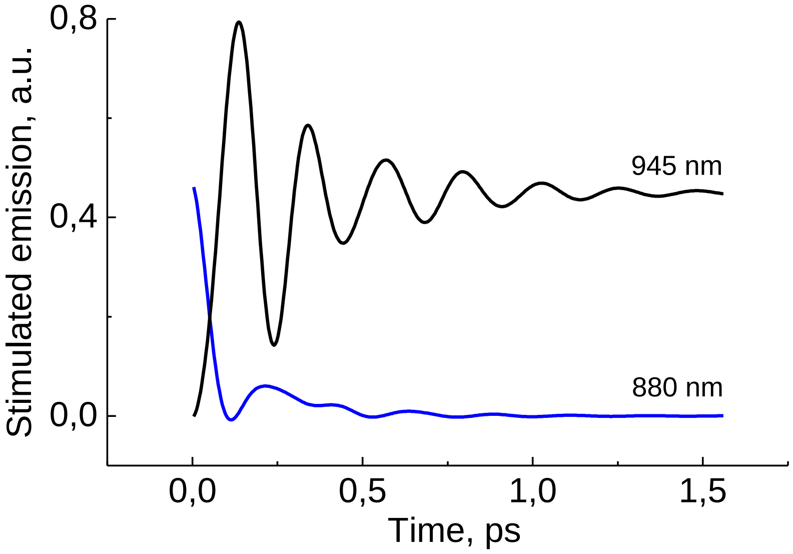


**Figure S4.** As Figure S2 but with initial vibrational coherence in the both $P_1$* and $P_2$* states. For details of the other parameters, see the main text and the legends in Figures S1, S2 and 4.

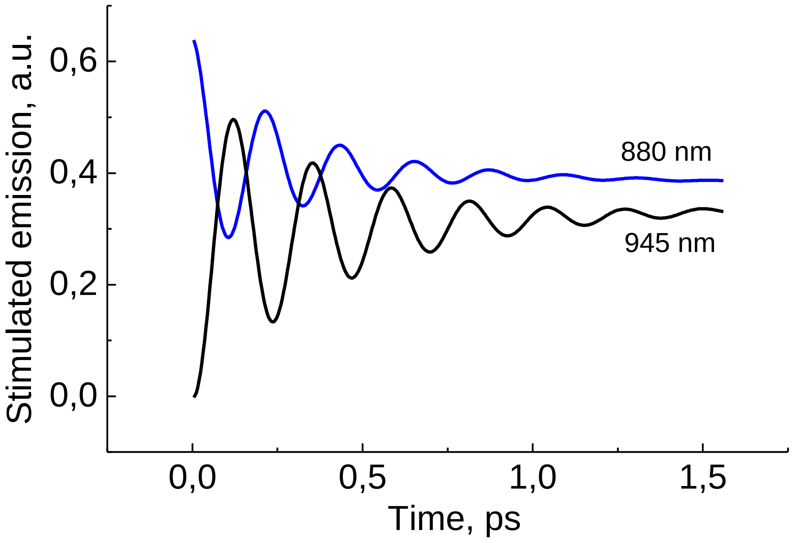


**Figure S5.** As Figure S2 but with $\Delta_1 = 1$. In this case the $P_1$* and $P_2$* terms are shifted in the same direction with respect to the $P_g$ term. For details of the other parameters, see the main text and the legends in Figures S1, S2 and 4.

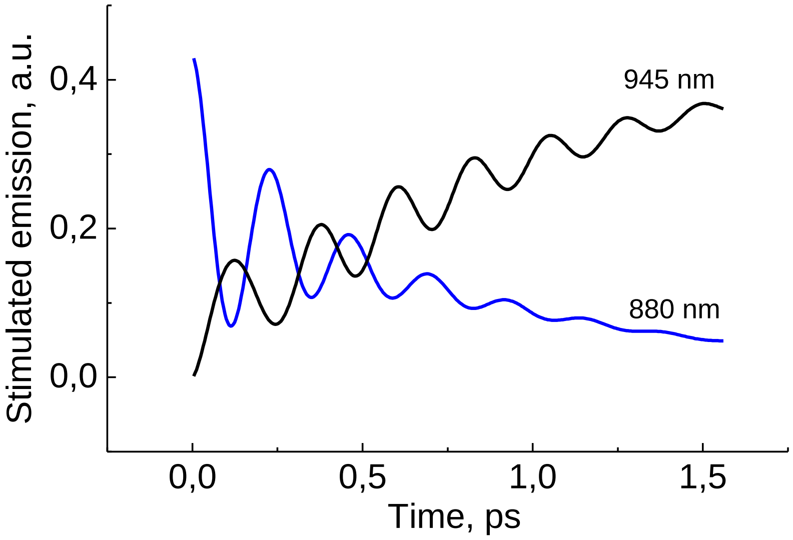


**Figure S6.** As Figure S2 but with smaller electronic coupling $J = 40\ \mathrm{cm}^{-1}$. For details of the other parameters, see the main text and the legends in Figures S1, S2 and 4.

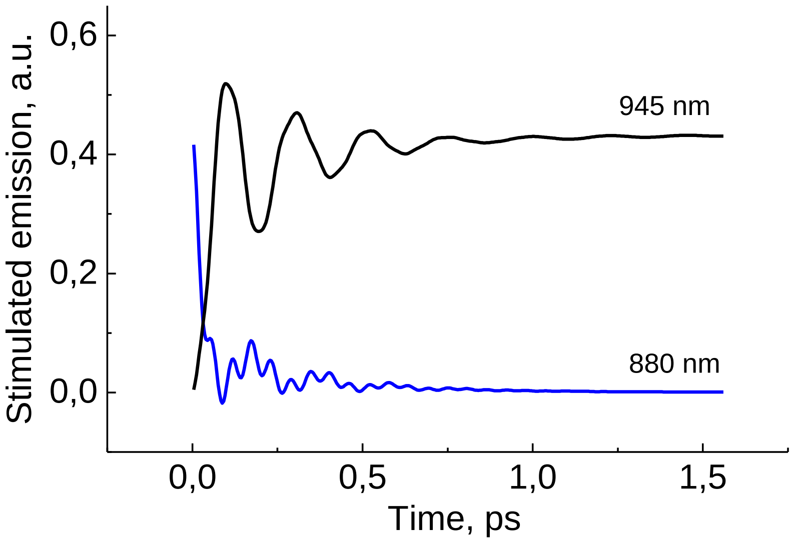


**Figure S7.** As Figure S2 but with greater electronic coupling $J = 800\ \mathrm{cm}^{-1}$. For details of the other parameters, see the main text and the legends in Figures S1, S2 and 4.

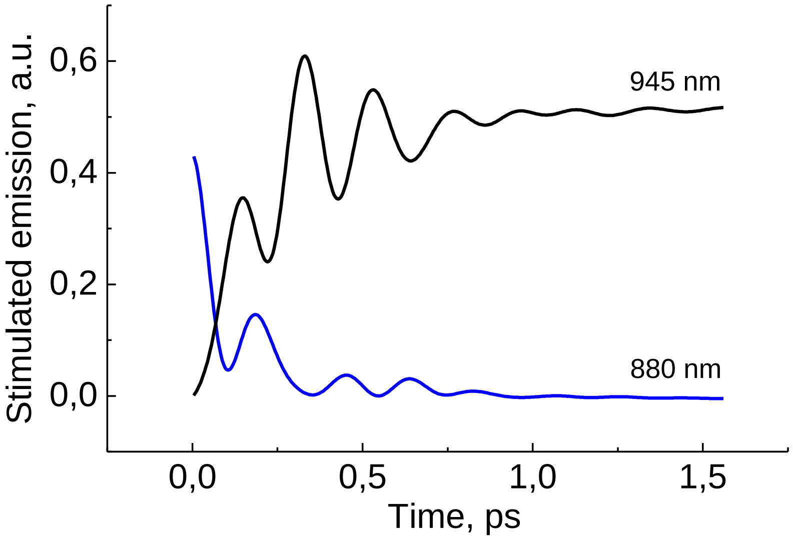


**Figure S8.** As Figure S2 but with smaller energy shift between the $P_1$* and $P_2$* terms, which is taken as $\hbar\omega$. For details of the other parameters, see the main text and the legends in Figures S1, S2 and 4.

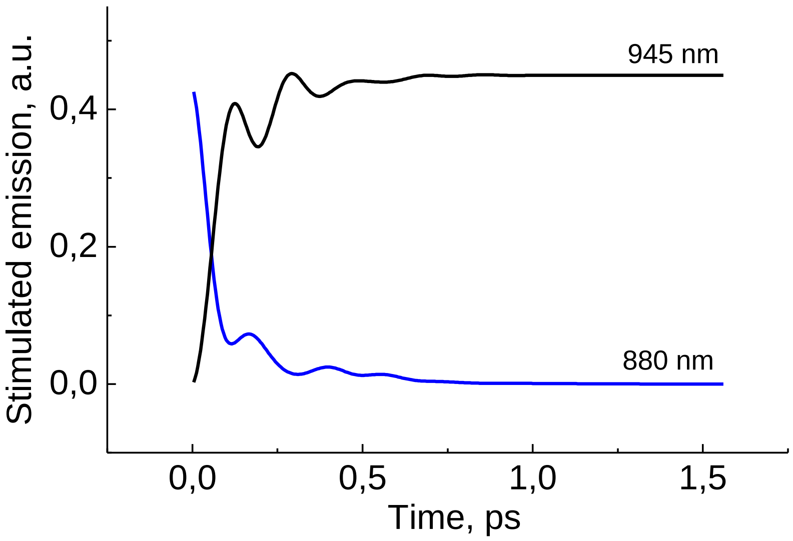


**Figure S9.** As Figure S2 but in the "secular" approximation. In this case, a vibronic coherence between non-neighboring levels and vertical coherence transfer are neglected.

## References


1. Vos, M.H., Jones, M.R., Breton, J., Lambry, J.C., Martin, J.-L. 1996. Vibrational dephasing of long- and short-lived primary donor excited states in mutant reaction centers of *Rhodobacter sphaeroides.* Biochemistry 35, 2687-2692.

2. Yakovlev, A.G., Vasilieva, L.G., Shkuropatov, A.Ya., Bolgarina, T.I., Shkuropatova, V.A., Shuvalov, V.A. 2003. Mechanism of charge separation and stabilization of separated charges in reaction centers of *Chloroflexus aurantiacus* and of YM210W(L) mutants of *Rhodobacter sphaeroides* excited by 20 fs pulses at 90 K. J. Phys. Chem. A**,** 107, 8330–8338.

3. Vos, M.H., Jones, M.R., Hunter, C.N., Breton, J., Lambry, J.-C., Martin, J.-L. 1994. Coherent dynamics during the primary electron-transfer reaction in membrane-bound reaction centers of *Rhodobacter sphaeroides*. Biochemistry 33, 6750-6757.

4. Lin, S.H. 1974. On the master equation approach of vibrational relaxation in condensed media. J. Chem. Phys. 61, 3810-3820.

5. Fain, B., Lin, S.H., Hamer, N.J. 1989. Two-dimensional spectroscopy: Theory of nonstationary, time-dependent absorption and its application to femtosecond processes. J. Chem. Phys. 91, 4485-4494.

6. Gu, X.Z., Hayashi, M., Suzuki, S., Lin, S.H. 1995. Vibrational coherence and relaxation dynamics in the primary donor state of the mutant reaction center of *Rhodobacter capsulatus*: Theoretical analysis of pump-probe stimulated emission. Biochim. Biophys. Acta. 1229, 215-224.

7. Lin, S.H., Hayashi, M., Suzuki, S., Gu, X., Xiao, W., Sugawara, M. 1995. Theoretical analyses on femtosecond time-resolved spectra of initial electron transfer of photosynthetic reaction centers at low temperatures. Chem. Phys. 197, 435-455.

8. Sugawara, M., Hayashi, M., Suzuki, S., Lin, S.H. 1996. Theoretical wave packet study on pump-probe stimulated emission signals from electron transfer systems in condensed phases. Mol. Phys. 87(3), 637-650.

9. Hayashi, M., Yang, T.-S., Chang, C.H., Liang, K.K., Chang, R.-L., Lin, S.H. 2000. Application of the density matrix method to spectroscopy and dynamics of photosynthetic reaction centers. Int. J. Quantum Chem. 80, 1043-1054.

10. Redfield, A. G. 1965. The theory of relaxation processes. In: *Advances in Magnetic Resonance,* Waugh J.S., (ed.), Acad. Press, New York, v. 1, pp.1-32.

11. Manneback, C. 1951. Computation of the intensities of vibrational spectra of electronic bands in diatomic molecules. Physica Grav. 17, 1001-1010.
